# Pristine microstructures in pseudotachylytes formed in dry lower crust, Lofoten, Norway

**DOI:** 10.1098/rsta.2019.0423

**Published:** 2021-02-01

**Authors:** Kristina G. Dunkel, Luiz F. G. Morales, Bjørn Jamtveit

**Affiliations:** 1 Physics of Geological Processes (PGP), The Njord Centre, University of Oslo, PO Box 1048 Blindern, 0136 Oslo, Norway; 2 Scientific Center for Optical and Electron Microscopy (ScopeM), ETH Zürich, Otto-Stern-Weg 3, 8093, Zürich; 3 Geological Institute, Department of Earth Sciences, ETH Zürich, Sonneggstrasse 5, 8092 Zurich, Switzerland

**Keywords:** earthquakes, lower crust, feldspar, spherulite

## Abstract

Feldspar-rich pseudotachylytes from the island of Moskenesøya, Lofoten, formed in dry granulites under lower crustal conditions during the Caledonian orogeny. The central parts of the pseudotachylytes, where the cooling rates were slowest, are characterized by microlites and spherulites of plagioclase and K-feldspar. K-feldspar surrounding plagioclase is consistent with crystallization from a melt during cooling instead of devitrification as the origin of the spherulites. Very thin (a few micrometres wide) injection veins, which experienced very rapid quenching, contain amorphous or cryptocrystalline material. The preservation of this material and of the fine-grained microstructures shows that, under fluid-absent conditions, recrystallization and reactions are slow and the original microstructures of the pseudotachylytes can be preserved.

This article is part of a discussion meeting issue ‘Understanding earthquakes using the geological record’.

## 1. Introduction

Seismic faulting can lead to frictional melting and the formation of pseudotachylytes: dark veins with a glassy to finely crystalline matrix. Pseudotachylytes are most common on faults in the seismogenic regime of the upper and middle crust [1], but have also been found in exhumed lower crustal rocks where they are taken as evidence for intermediate-depth earthquakes (e.g. [2–9]). The origin of these earthquakes is somewhat enigmatic because at the high temperatures and confining pressures in the lower crust, deformation would be expected to be viscous (e.g. [10]). To overcome the high confining pressure and allow for frictional failure, stresses have to be very high or local weakening mechanisms must be active. Sufficiently high stresses cannot be sustained over orogenic timescales and would have to be transient, potentially caused by earthquakes in the shallower crust [11]. In the absence of high stresses, possible weakening mechanisms include thermal runaway [12,13], plastic instabilities [9,14] and dehydration- or reaction-induced embrittlement (e.g. [15]).

Pseudotachylytes originating at all depths are rare in the rock record compared to the expected number of earthquakes [1]. One possible reason for this observation is the presence of fluid in most fault zones: Sibson & Toy [1] suggested that only dry, intact, crystalline rocks produce pseudotachylytes. By contrast, when fluids are present in a fault, thermal pressurization of those fluids during seismic slip would inhibit melt generation. Another explanation for the scarcity of pseudotachylytes is that their fine-grained or amorphous nature makes them susceptible to deformation, recrystallization and alteration, so that they are easily overlooked in the rock record [16]. The introduction of fluids during or after faulting in particular causes alteration of the mineral assemblage in the pseudotachylyte. In the absence of fluids and deformation, pristine pseudotachylyte microstructures can be preserved. This has been the case for pseudotachylytes in feldspar-rich rocks on Moskenesøya in Lofoten, Northern Norway. The microstructures of the Moskenesøya pseudotachylytes, which formed under lower crustal conditions during the Caledonian orogeny, are remarkably well preserved. A general description of these pseudotachylytes has been provided by Dunkel *et al*. [17], who interpreted them to be the result of transiently high stresses in the lower crust based on the absence of ductile deformation and the anhydrous mineralogy (see §3b). Here, we describe the microstructures of the pseudotachylytes in detail via scanning electron microscopy (SEM) and transmission electron microscopy (TEM) and associated analytical techniques and discuss their development.

## 2. Methods

Thin sections were prepared perpendicular to the faults that show pseudotachylyte veins.

A Hitachi SU5000 FE-SEM (Field Emission Scanning Electron Microscope; Department of Geosciences, University of Oslo) was used for backscatter electron imaging and electron backscatter diffraction (EBSD) analysis. Crystallographic orientations of the minerals in the samples were determined by indexing of EBSD patterns, as acquired with a Bruker e-Flash detector. For EBSD mapping, the FE-SEM was used at low vacuum with uncoated samples, an accelerating voltage of 20 kV and a working distance of 20 mm. The step size used for EBSD mapping was 0.3 µm. EBSD patterns were indexed automatically using the Esprit v. 2.1 software from Bruker. Qualitative energy-dispersive spectrometry (EDS) element maps, which were acquired concurrently to the EBSD maps, were used to assist the phase identification. Visualization of the crystallographic orientation, calculations of the orientation distribution functions (ODFs) and plotting of pole figures were conducted using the Matlab toolbox MTEX (v. 5.5.1; http://mtex-toolbox.github.io) [18–20]. For the ODF calculations, the de la Vallée Poussin standard orientation distribution function was used, with a halfwidth of 10°.

Three *in situ* TEM lamellae were prepared using a Helios 600i focused ion beam (FIB)-SEM machine. Initially, a 15 × 2 × 2 µm (length × width × thickness) layer of platinum was deposited on top of the region of interest using the FIB operating at 30 keV and a beam current of 300 pA. The frontal and back trenches were created with the Ga-FIB operating at 30 keV and 15 nA beam current, followed by a cleaning step at 3 nA. The lamellae were thinned down using beam currents of 300, 150 and 80 pA, up to a final thickness of 100 nm. The TEM images were obtained in a Hitachi HT7700 EXALENS and an FEI Tecnai F30 FEG, both in operation at the Scientific Center for Optical and Electron Microscopy of ETH Zürich. EDS analyses were conducted at 200 kV, with the interaction depth corresponding to the thickness of the sample.

## 3. Background

### (a) Geological setting

The Lofoten-Vesterålen islands are part of the Norwegian Caledonides and consist of an Archean to Paleoproterozoic metamorphic complex of para- and orthogneisses. This sequence was intruded under granulite-facies conditions at approximately 1870–1770 Ma by an Anorthosite–Mangerite–Charnockite–Granite (AMCG) suite that makes up about 50% of the archipelago [21,22].

During the Caledonian orogeny (*ca* 490–390 Ma), western Baltica was partially subducted under Laurentia; however, the relation of the Lofoten block to Baltica and the Caledonides is unclear. In Early Carboniferous plate reconstructions, where east Greenland is welded to the Norwegian margin, Lofoten has a central location in the northern parts of the restored orogeny. It records very few Caledonian deformation structures [5], which is probably due to limited fluid availability and thus the high strength of the anhydrous granulites [23].

Pseudotachylytes generated at lower crustal conditions have been reported from numerous localities in Lofoten-Vesterålen [5–7,24–28]. Pseudotachylytes are often coeval with eclogite or amphibolite facies shear zones [5,6,25], indicating formation under lower crustal conditions.

### (b) Previous work

**Figure 1.**
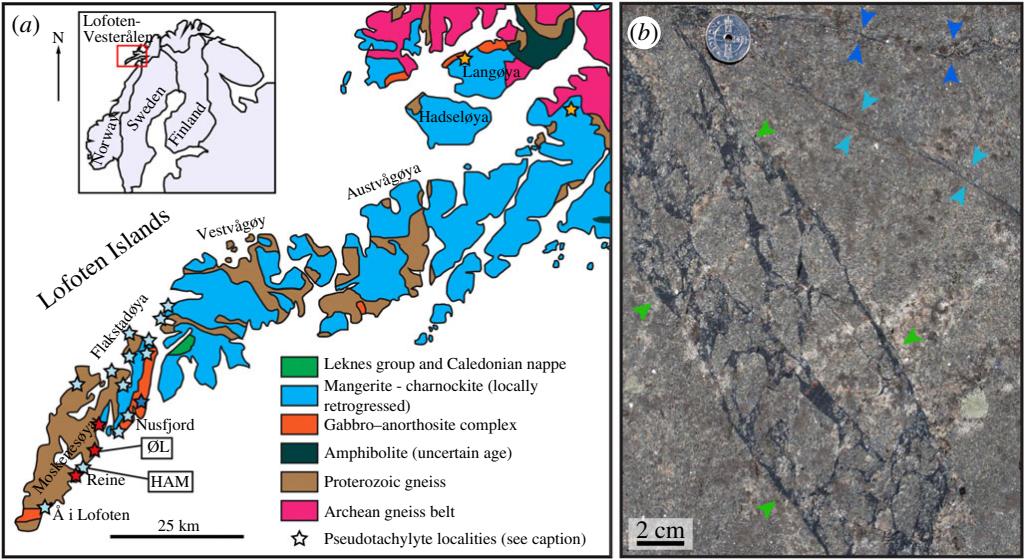
(*a*) Geological map of Lofoten (modified from [21]), with the positions of the sampling localities HAM and ØL on Moskenesøya. (HAM: 67.94915, 13.13722, ØL: 67.96589, 13.16921.) Pseudotachylyte localities are from Menegon *et al*. [6] (dark blue star), Steltenpohl *et al*. [26] (yellow), Jamtveit *et al*. [7] (light blue) and Dunkel *et al*. [17] (red). (*b*) Field photograph of pseudotachylytes in mangerite at locality HAM. Pseudotachylyte breccia between the green arrowheads, single pseudotachylyte veins between the sets of blue arrowheads. Photo courtesy of Luca Menegon.

The pseudotachylyte samples discussed in this study were collected at roadcuts along the east coast of Moskenesøya (figure 1*a*). The occurrence and chemical and mineralogical composition of these pseudotachylytes were described by Dunkel *et al*. [17]: they occur as thin (*ca* 1–15 mm), dark veins in massive, undeformed granulites of mangeritic and gabbroic composition. They often have injection veins or form pseudotachylyte breccias (figure 1*b*). The host rocks contain mainly plagioclase and K-feldspar, variable amounts of pyroxenes (orthopyroxene in the mangeritic, ortho- and clinopyroxene in the gabbroic varieties) and amphibole. Ti-bearing magnetite is common in both rock types. Small amounts of quartz are limited to the mangeritic rocks. The pseudotachylytes reflect the mineralogical composition of their hosts, with the addition of some garnet, which only occurs as rare overgrowths in the host rocks and is slightly more common in the pseudotachylytes.

Because this mineralogy did not allow for conventional geothermobarometry, the pressure conditions during pseudotachylytes formation were estimated using elastic geobarometry on quartz inclusions in garnet [17]. Other, hydrous pseudotachylytes and shear zones in Lofoten record temperatures of *ca* 650–750°C. Assuming similar temperatures for the Moskenesøya pseudotachylytes, pressures were in the range 0.8–1.2 GPa, indicating lower crustal formation conditions for the pseudotachylytes. Additionally, the absence of hydrous phases alone suggests that faulting did not occur in the shallow crust, where fluid infiltration usually accompanies or follows an earthquake.

Neither the host nor the clasts in the pseudotachylyte show evidence of pre-seismic ductile deformation. Therefore, Dunkel *et al*. [17] excluded thermal runaway or plastic instabilities as possible mechanisms for the formation of these lower crustal earthquakes. Dehydration embrittlement is not considered a viable mechanism either because of the lack of biotite, which would have formed under hydrous conditions. In the absence of such weakening mechanisms, Dunkel *et al*. [17] suggested transiently high stresses in the dry, strong granulite-facies rocks.

## 4. Results

All mineral phases present in the host rock (plagioclase, K-feldspar, pyroxene, magnetite, amphibole, quartz, see §3b) also occur as angular to subrounded lithoclasts in the pseudotachylyte. The main newly grown phases in the pseudotachylyte matrix are plagioclase, K-feldspar, garnet and magnetite. In the following, we describe the micro- and nanostructures of those phases in detail.

### (a) Pseudotachylyte microstructures

**Figure 2.**
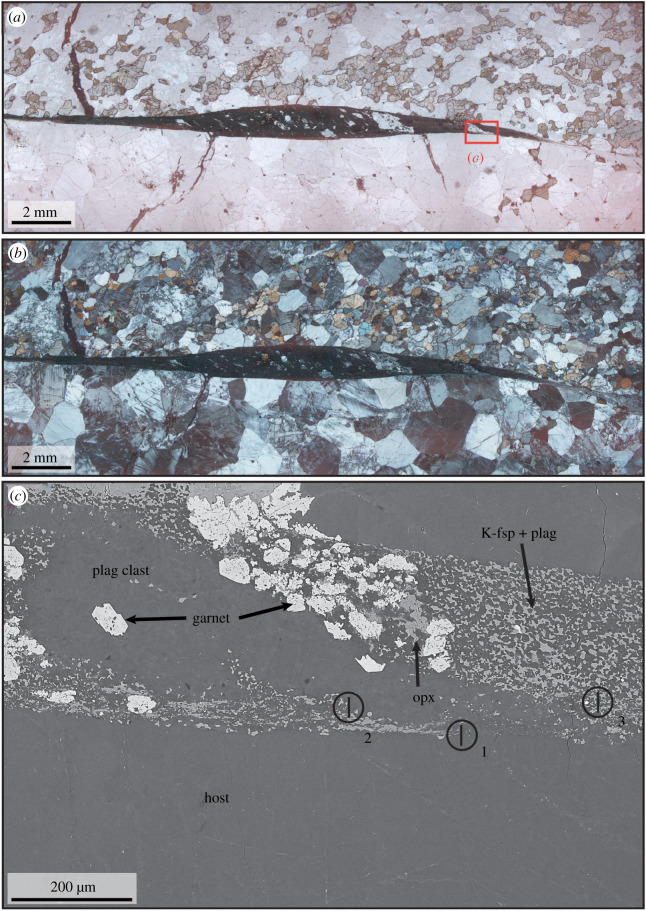
(*a,b*) Optical photomicrographs of sample ØL-1A with a long, straight pseudotachylyte vein with several injection veins in plane- and cross-polarized light, respectively. The colourless phase is predominantly plagioclase, the pale pink and pale green grains are pyroxenes and the more strongly green grains amphibole. The red rectangle in subfigure (*a*) marks the position of subfigure (*c*). (*c*) Electron backscatter image from the pseudotachylyte vein, showing the location of the TEM lamellae 1–3 displayed in figures 6–9. The plagioclase clast is heavily fragmented, as evidenced by the growth of garnet in its middle.

Representative samples from two localities are shown in figures 2 (locality ØL) and 3 (HAM). The feldspars exhibit a variety of fine-grained microstructures in the pseudotachylyte matrix (see below), but some large feldspar clasts (diameters on the order of 100 µm) have been observed as well. Magnetite occurs mainly as small grains (diameter of a few micrometres). Rare larger grains have diameters comparable to the magnetite grains in the host rock. Pyroxene and amphibole occur as small angular to slightly embayed clasts (*ca* 10 µm in diameter). Quartz is rare and has been observed as both larger clasts and as part of the pseudotachylyte matrix. Garnet has a dendritic to snowball habit with occasional euhedral overgrowths (diameter few 10 s of µm), with inclusions of the other pseudotachylyte phases (figure 2*c*).

**Figure 3.**
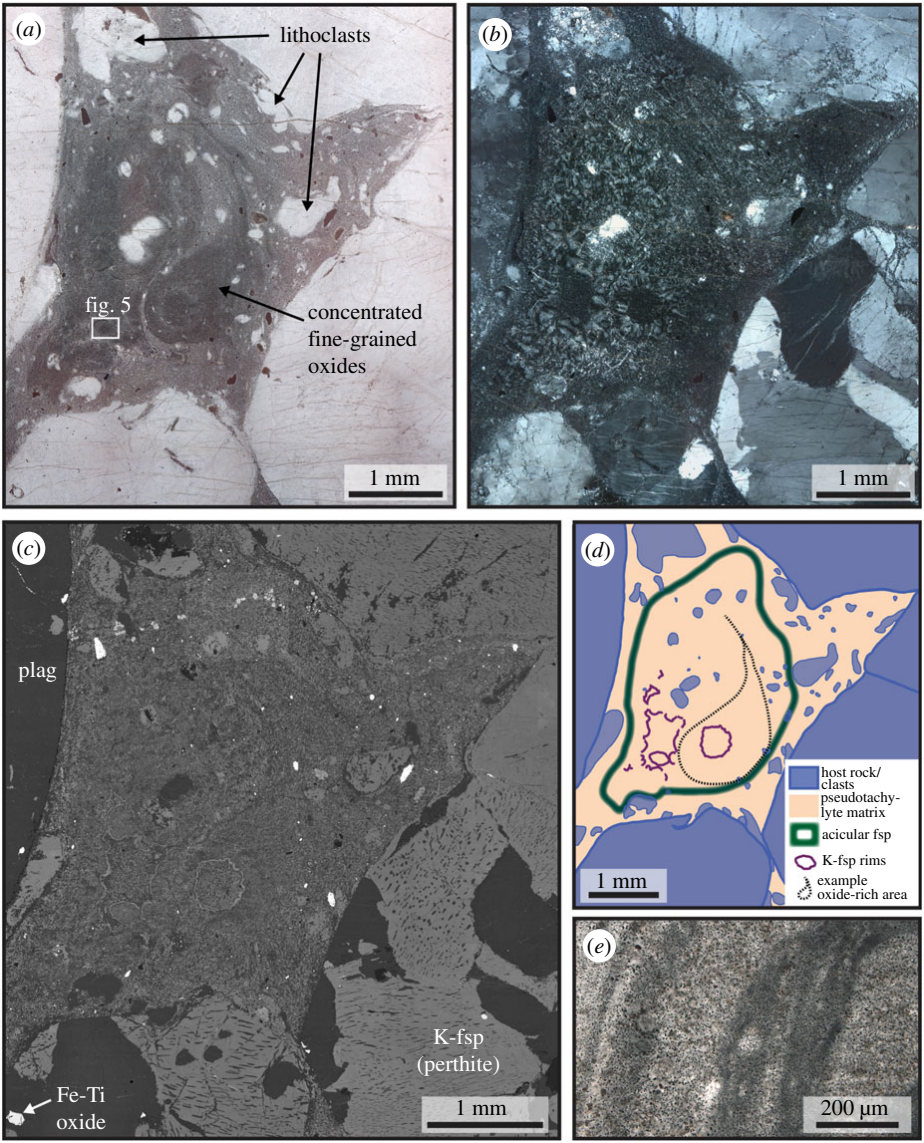
Microstructures of pseudotachylyte pockets. (*a–d*) Sample HAM-2C. (*e*) Sample HAM-3C. (*a,b*) Optical photomicrograph in plane- and cross-polarized light, respectively. The darker areas in (*a*), which are rich in fine-grained Fe-oxide, show a distribution resembling flow structures. The feldspar microstructures in the same area, however, are spherulitic without noticeable deformation, as visible in cross-polarized light (*b*). (*c*) Backscatter electron image of the same area. The clasts in the pseudotachylyte are not elongated. (*d*) Sketch of the area depicted in (*a*) to (*c*), showing how the acicular feldspar (microlites and spherulites) is limited to the centre of the melt pocket. Note also the outline of an area rich in magnetite with a flow structure (see (*a*)) within the domain of undeformed acicular feldspar. (*e*) Dark stripes rich in fine-grained magnetite in the pseudotachylyte.

**Figure 4.**
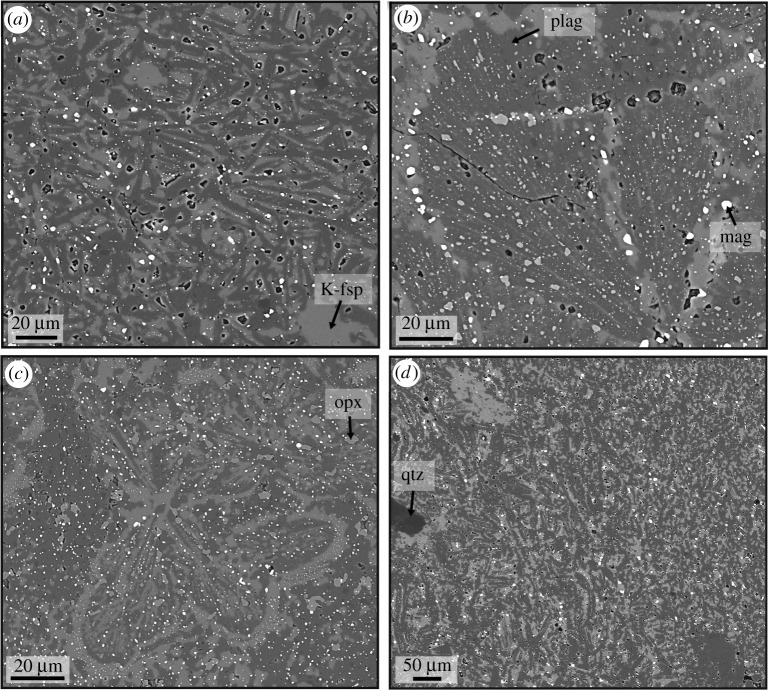
Feldspar microstructures. (All four electron backscatter images are taken at comparable settings, so that the phase labels apply to all subfigures.) (*a*) Plagioclase microlites of variable orientation. (*b*) Sheaf microlite of plagioclase. (*c*) Bundles of plagioclase and K-feldspar microlites surrounded by K-feldspar rims with a high density of small magnetite inclusions. (*d*) Transition from microlitic plagioclase surrounded by K-feldspar (bottom left) to an irregular mix of both feldspars (top right).

**Figure 5.**
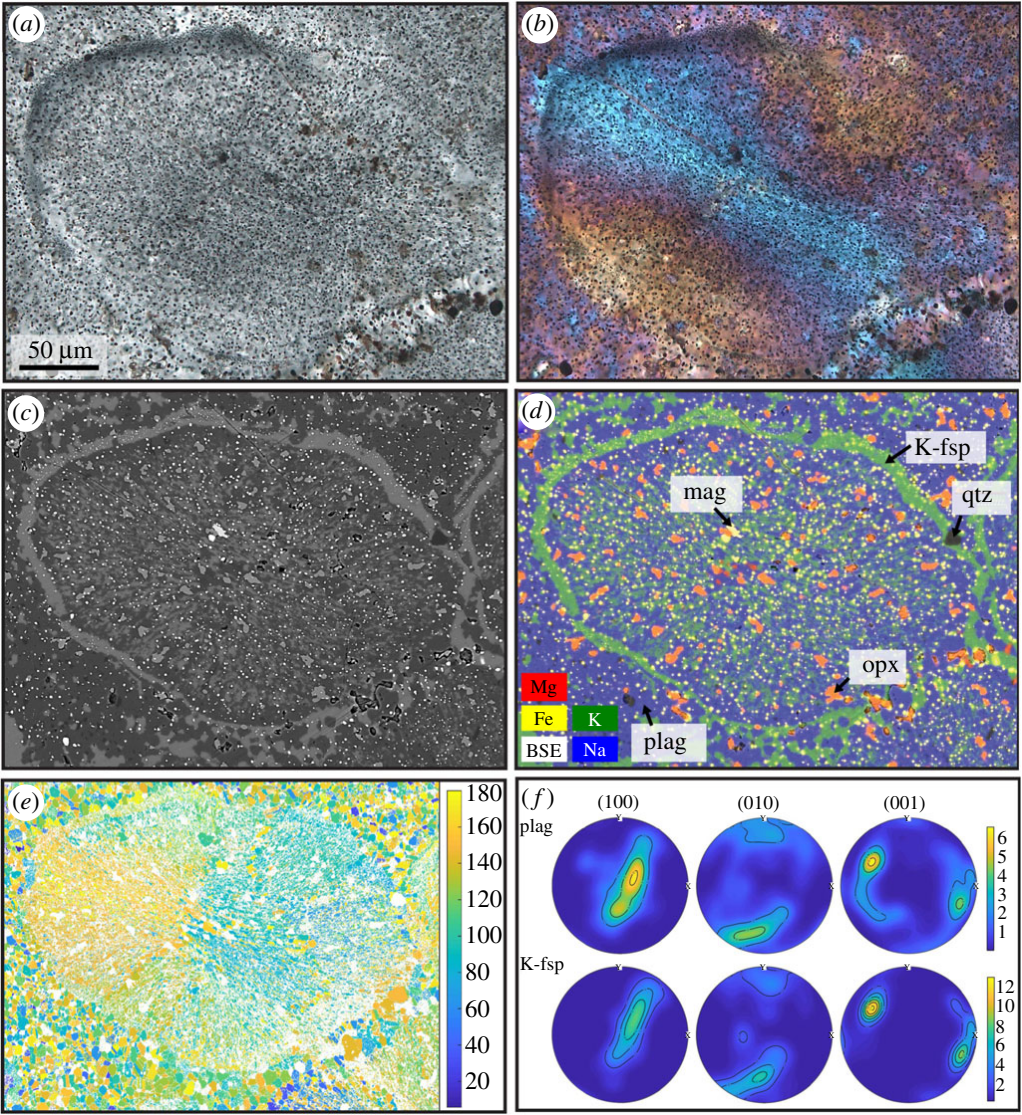
Spherulite consisting mainly of an intergrowth of feldspars, sample HAM-2C. See figure 3*a* for the location of this spherulite in the pseudotachylyte. (*a*) Optical photomicrograph in plane-polarized light. (*b*) Optical photomicrograph in cross-polarized light, with the λ-plate inserted, showing a radial distribution of orientations. (*c*) Backscatter electron image and (*d*) combined element distribution maps of the same area, showing the fine-grained nature of the spherulite and the rim of K-feldspar surrounding the spherulite. (*e*) Combined orientation map for plagioclase and K-feldspar, with a random reference orientation. (*f*) Pole figures for plagioclase and K-feldspar (for every pixel within the spherulite domain), showing the consistent orientation of the two feldspars.

Sample ØL-1A contains a pseudotachylyte vein with a maximum thickness of *ca* 2 mm. Injection veins branch off at high angles. The fault vein contains elongated clasts (figure 2), which are heavily fragmented (figure 2*c*). The pseudotachylyte matrix consists of equigranular plagioclase and K-feldspar. In thicker fault veins and especially in injection veins and irregularly shaped melt pockets (e.g. sample HAM-2C, figure 3), there is a large variety of microstructures, largely determined by the habit of the two feldspars, which always occur together and constitute most of the pseudotachylyte matrix. The two feldspars occur as equant grains or, especially in the case of plagioclase, elongated microlites. The microlites can be single, randomly oriented grains (figure 4*a*), form bundles of parallel microlites (figure 4*b,c*) or occur as spherulites (figure 5). In some pseudotachylyte veins, a zonation with microlites/spherulites in the centres and equant grains towards the edges can be observed (figures 3*d* and 4*d*). However, in other pseudotachylytes, no such zonation is visible, which might be due to the complex shape of the pseudotachylyte network.

In the microlitic or spherulitic domains, plagioclase laths are often surrounded by K-feldspar (figure 4*a,d*). In some cases, however, the two feldspars are intergrown and the microlite bundles or spherulites as a whole are rimmed with K-feldspar (figures 4*c* and 5*c*). Even though the spherulites do not consist of single, elongated crystals radiating outwards from a centre, but of an intergrowth of plagioclase and K-feldspar (figure 5*c,d*), the feldspars show a radial distribution of their crystallographic orientation, as expected from spherulites, in cross-polarized light (figure 5*b*). This radial distribution was confirmed by measuring the crystallographic orientations with electron backscatter diffraction (figure 5*e*). The orientations of neighbouring plagioclase and K-feldspar are subparallel (figure 5*e,f*).

Magnetite grains occur finely dispersed throughout the pseudotachylyte matrix. Especially in some of the larger pseudotachylytes or melt pockets, magnetite grains are arranged in strings, indicating flow (figure 3*a,e*). In the same area, unstrained feldspar microlites and spherulites occur (figure 3*d*).

### (b) Pseudotachylyte nanostructures

Three TEM lamellae were made from sample ØL-1A. Lamella 1 is at the boundary between host and pseudotachylyte, lamella 2 between pseudotachylyte and a plagioclase clast, and lamella 3 is within the pseudotachylyte (figure 2*c*).

**Figure 6.**
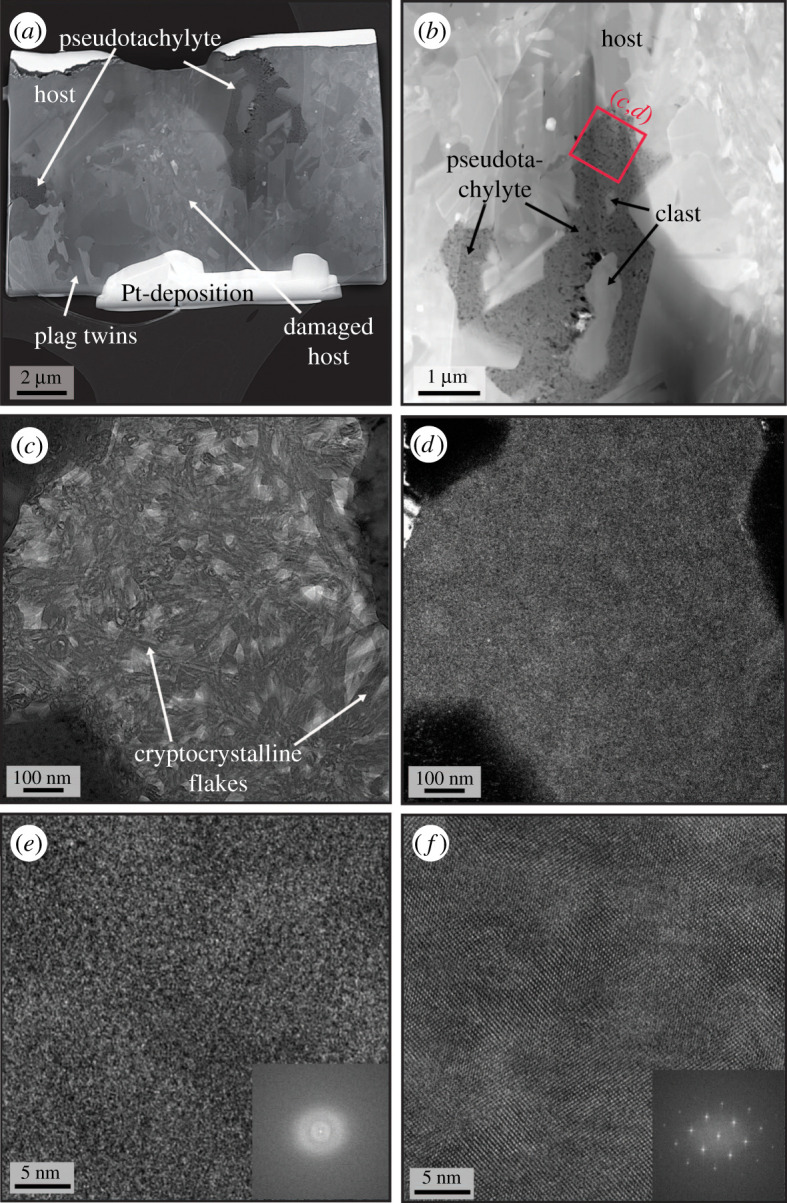
TEM images of lamella 1, marked in figure 2*c*. (*a*) High-angle annular dark-field (HAADF) overview of the full lamella, showing the granulite host, comprising large, twinned plagioclase grains, with a pocket of the darker pseudotachylyte material. (*b*) The pseudotachylyte (dark area) seems to occur along intragranular fractures in the host plagioclase and contains lithoclasts. (*c*) Bright-field image from the pseudotachylyte, showing amorphous material mixed with cryptocrystalline material. (*d*) Dark-field images of the same area as (*c*), in which the pseudotachylyte appears predominantly homogeneous, suggesting the dominance of amorphous material in this region. (*e,f*) High-resolution TEM (HRTEM) image from the melt pocket (*e*) and the lithoclast (*f*) and the respective fast Fourier transforms (insets) show the amorphous nature of the material in the pseudotachylyte and the standard crystallinity of plagioclase.

**Figure 7.**
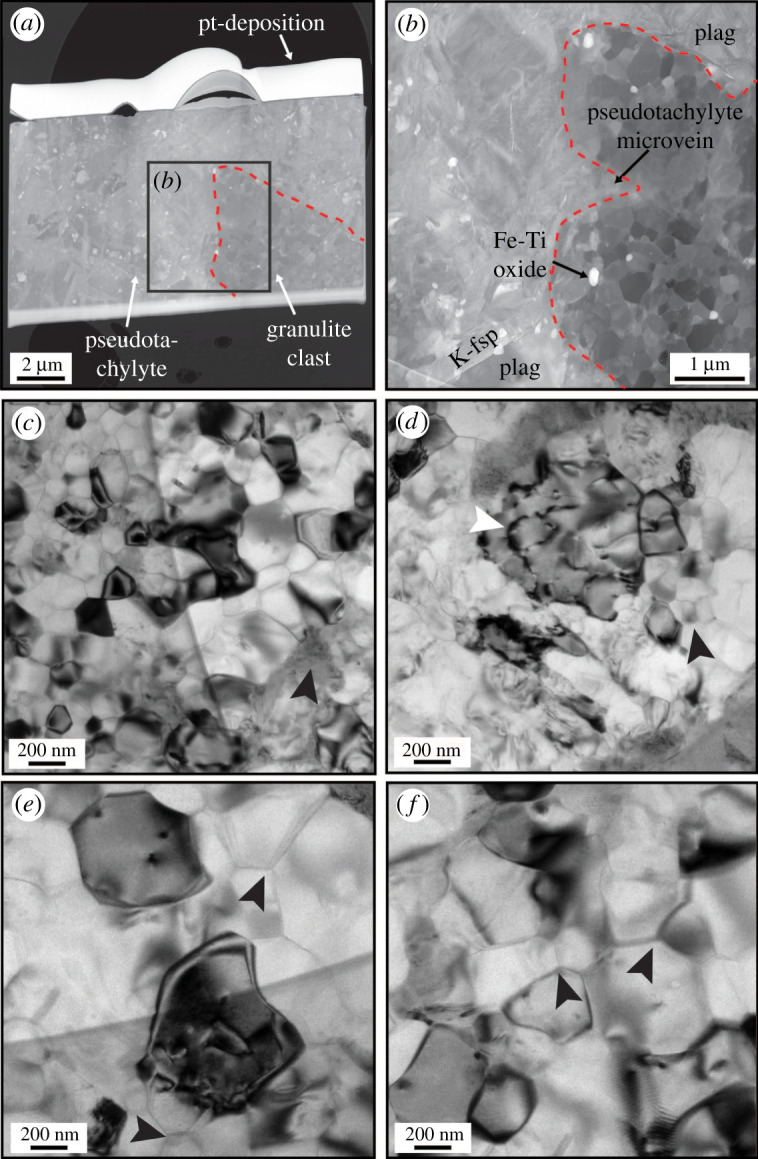
TEM images of lamella 2 marked in figure 2*c*. (*a*) High-angle annular dark-field (HAADF) overview of the full lamella, showing a granulite clast and the pseudotachylyte vein. (*b*) HAADF image showing a detail of subfigure **(***a*) with a pseudotachylyte microvein intruding the granulite clast (on the right), and crystals of plagioclase, K-feldspar, and nanocrystals of Fe-Ti-oxide. Note the sharp grain boundaries in the granulite clast. (*c–f*) TEM bright-field images from the plagioclase-dominated granulite clast. Most of the grains are inequigranular and strain free, showing predominant straight, tight grain boundaries (*c*), in many places forming triple and quadruple junctions with no porosity along the interfaces (*e–f*). Note that some of the grains have visual grain sizes of approximately 50 nm. A small amount of pseudotachylyte along one of the grain boundaries is marked by the arrowhead in (*c*). ‘Larger’ plagioclase crystals have cells of relatively high dislocation density (*d*), locally organized in subgrain walls (white arrowhead in (*d*)), and it is not clear whether this is inherited from previous ductile deformation or related to the damage zone along the slip interface. Note however that the subgrain size marked by the white arrowhead in *d* is similar to the ‘recrystallized’ grains (black arrowhead) surrounding it. Some grains in subfigures (*c–f*) appear dark because they are in strong diffraction position.

In lamella 1, spanning the granulite host and the pseudotachylyte vein, the plagioclase crystals are large and seem to preserve their original texture (figure 6*a*), with magmatic textures and twins and no clear evidence of grain size reduction as observed in figure 7. There is a small pocket of pseudotachylyte material within this sample, which wraps around a small plagioclase lithoclast that has embayed boundaries to the pseudotachylyte material (figure 6*b*). The diffraction contrast in bright-field imaging (figure 6*c*) indicates the presence of crystalline, flake-like particles. The weak dark-field image contrast acquired with one of the fundamental reflections from the same area (figure 6*d*) is due to the insufficient volume fraction of the crystalline phase in the area. The high-resolution TEM micrographs and corresponding fast Fourier transforms evidence the presence of both amorphous and crystalline phases in the specimen (figure 6*e,f*). Since the TEM instrument used for the studies does not feature aberration correction, it is no possible to exclude that the amorphous phase is in reality a cryptocrystalline phase.

**Figure 8.**
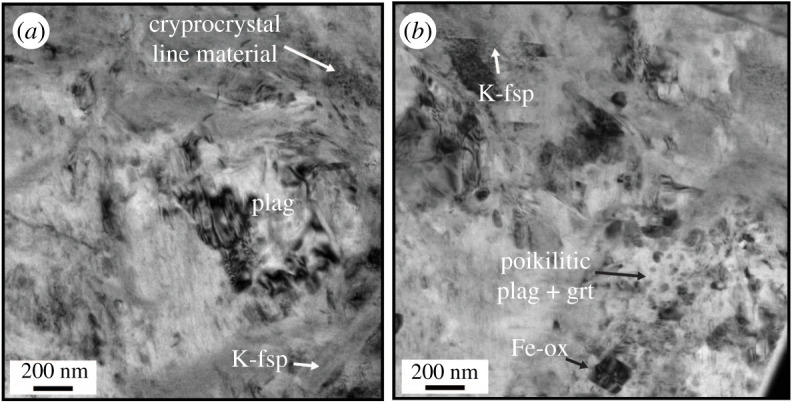
Bright-field TEM images of the pseudotachylyte portion of lamella 2 marked in figure 2*c*. (*a*) Possible remnant plagioclase with relatively high dislocation density and ‘nanolites’ of K-feldspar crystallized within the pseudotachylyte and some cryptocrystalline material. (*b*) Poikilitic crystals of plagioclase and mainly garnet within an amorphous matrix, together with Fe-Ti-oxide, nanolites of K-feldspar and plagioclase. The ‘crispy’ aspect of the images is partially due to beam damage.

The high-angle annular dark-field (HAADF) overview of the second lamellae (figure 7*a*) contains a lithoclast, but is dominated by the pseudotachylyte. The clast is composed primarily of plagioclase (with minor amounts of Ti-rich magnetite) and is damaged, as demonstrated by the intense grain size reduction observed in the plagioclase, with grain sizes varying from 200 to 1000 nm (figure 7). The bright-field images from the damaged host rock show that the plagioclase grains are predominantly free of dislocations and have straight grain boundaries (figure 7*c*), and in a number of places, triple and quadruple junctions can be observed (figure 7*c–f*). Although not common, larger plagioclase crystals have high dislocation density cells, which can be partially organized in subgrain walls with similar dimensions to the recrystallized grains just adjacent to the large grain (figure 7*d*). Nanometre-wide pseudotachylyte veins intruded the polycrystalline host and partially corroded the plagioclase in contact with the melt (figure 7*c*). In the bright-field TEM mode, plagioclase (figure 8*a*) and the small Fe-Ti oxides in the pseudotachylyte (figure 8*b*) display some dislocations, as shown by the variable contrast, whereas K-feldspar is largely strain free. Locally, nanometre-scale rounded crystals of plagioclase and garnet occur within an amorphous portion of the TEM lamella (figure 8*b*), which has a similar composition to point 4 in figure 8.

**Figure 9.**
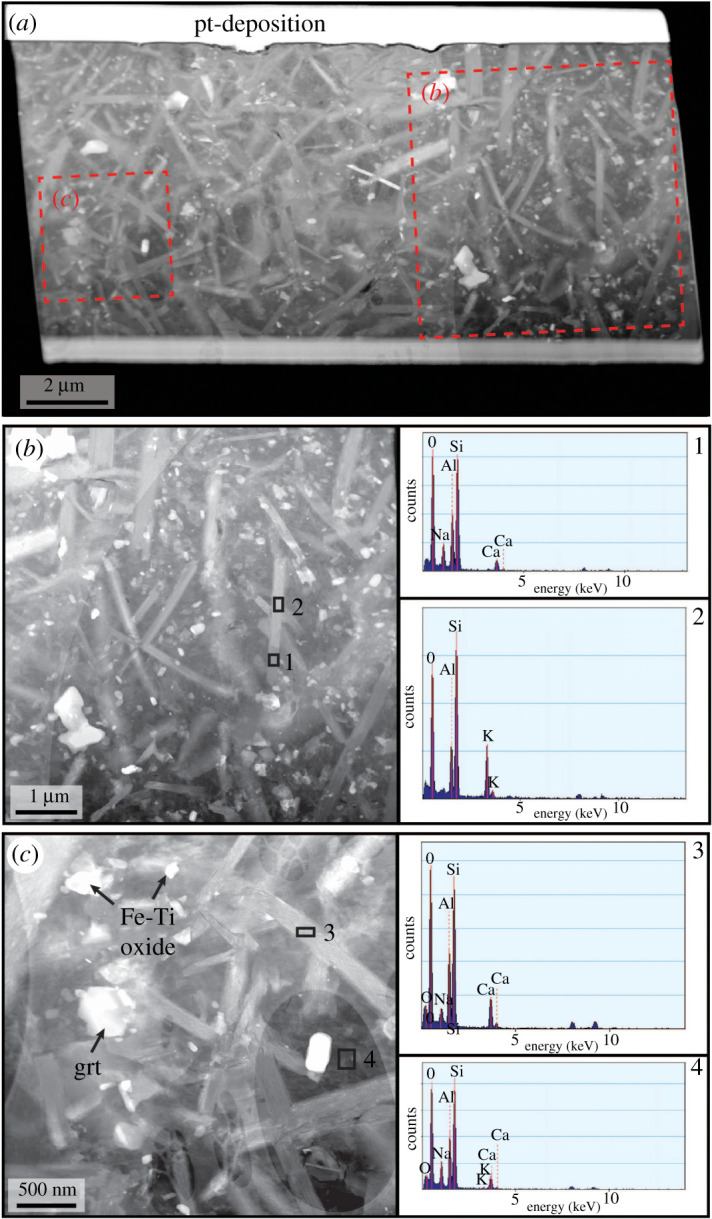
TEM images and compositional data from lamella 3, marked in figure 2*c*. (*a*) High-angle annular dark-field (HAADF) overview of the full lamella. (*b,c*) Detailed HAADF images from the pseudotachylyte vein, showing microlites of plagioclase and K-feldspar, garnet and an abundance of Fe-Ti oxides. On the right side, EDS point analyses of some of the common phases present in the pseudotachylyte are shown. Note that the little peaks of Ga and Cu are not related to the sample, but due to FIB sample preparation (Ga) and the TEM lamellae grid (Cu). An acicular feldspar in (*b*) is divided, perpendicular to its long axis, into plagioclase (point 1) and K-feldspar (2). Point 3 shows another plagioclase analysis; 4 is an analysis of the matrix between the acicular crystals, which has an alkali-feldspar-like composition.

EDS analysis of the pseudotachylyte veins from TEM lamella 3 show plagioclase (figure 9, points 1 and 3) and K-feldspar (figure 9, point 2) microlites/nanolites, with aspect ratios on the order of 5 : 1 and no clear preferred orientation relative to the vein walls (figure 9*a–c*). Ti-rich magnetite with grain sizes from 100 to 400 nm is also common in the pseudotachylytes (figure 9*c*). The matrix has a K-feldspar-like composition (figure 9, point 4).

## 5. Discussion

In the following, we will interpret the evolution of the studied fault rocks over the very short time interval from brittle failure to complete solidification of the frictional melt based on the petrographic observations described above and discuss the implications of the preservation of the described microstructures.

### (a) Fragmentation, melting and flow

During dynamic rupture and subsequent frictional slip, the host rock was fragmented, producing the clasts of feldspar, quartz, magnetite, pyroxene and amphibole that are later preserved as lithoclasts in the pseudotachylyte (e.g. Figure 3*a–c*). Frictional melting led to the formation of a melt rich in feldspar components. Parts of the melt that formed along the main fault surface intruded into the damaged wall rock, forming injection veins and melt pockets. During this process, flow structures, in particular the alignment of magnetite grains (figure 3), were produced in the particle-laden melt. The magnetite grains revealing the flow structures (figure 3*a*) are surrounded by microlitic, randomly oriented feldspar grains, showing that the flow took place in the molten state, before the crystallization of the feldspars, and not by crystal-plastic deformation after the solidification of the pseudotachylyte.

### (b) Crystallization of feldspar—the role of nucleation rate and undercooling

Rapid quenching, especially in thin injection veins, prevented crystallization and resulted in the formation of amorphous or cryptocrystalline material (figure 6). Especially in the centres of larger pseudotachylytes, microlites and spherulites are common (figures 4 and 5). Spherulites are arrays of elongated crystals radiating from a common ‘core’ and are typical of felsic volcanic rocks, but also very common in pseudotachylytes [29]. While the standard view of spherulites is often single, acicular crystals radiating outwards from a common centre, there are a multitude of possible morphologies, including densely branched spherulites (e.g. [30]). We suspect that this is the case here and that many of the small feldspar grains visible in the spherulites (figure 5*c,d*) are connected in three dimensions. Their origin can be devitrification of glassy material or rapid crystallization during quenching. In the present case, rapid crystallization from a melt is the most probable mechanism because of the spatial relation between plagioclase and K-feldspar (see below).

During direct crystallization from a melt, the presence or absence of equant grains, single microlites and spherulites can be used as rough indicators of cooling rates, but those microstructures also depend on other factors such as composition and water content [31]. In general, large undercooling favours the formation of surface instabilities during crystal growth because of the associated low diffusivity and fast growth rate [32]. In experimental crystallization of plagioclase at 0.5 GPa, Lofgren [33] observed spherulitic growth at undercooling from 150°C to 400°C. However, those experiments were conducted under water-saturated conditions, whereas we assume that the melt was anhydrous (and at higher pressure).

The cooling rate was lower in the centres of the pseudotachylyte veins or pockets than at the edges, in contact with the wall rock. This scenario resulted in a higher degree of resorption of feldspar clasts in the vein centres, causing a decrease in the amount of wall rock fragments from vein margin to centre. The higher degree of undercooling in the outer parts of the veins also led to a higher nucleation rate. Combined with the higher number of clasts that can provide sites for heterogeneous nucleation, this resulted in the growth of many small, equigranular feldspar grains. However, the occurrence of nanolites observed in TEM in ØL-1A (figure 9) shows that structures indicative of fast crystallization can be present even when not visible at lower (SEM) magnification. What appears as equigranular grains in SEM observations may be aggregates of nanolites in a cryptocrystalline or amorphous matrix. Toward the centres of the pseudotachylytes, the nucleation rate and number of clasts was lower, while the growth rate was still high, leading to the formation of microlites and spherulites of plagioclase visible even in optical microscopy (figures 3*b* and 5*a,b*).

The intergrown plagioclase and K-feldspar spherulites rimmed by K-feldspar (figure 5*c*) indicate eutectic crystallization of both feldspars followed by the growth of pure K-feldspar. In other cases, K-feldspar occurs around plagioclase laths (figure 3*f*). K-feldspar surrounding plagioclase indicates the direct growth of the two feldspar phases from a melt, because this spatial separation is unlikely to have originated during devitrification. A similar separation of plagioclase and K-feldspar has been observed in pseudotachylytes formed in the Adamello tonalites under shallower conditions, with K-feldspar-rich rims around plagioclase-rich spherulites [34].

### (c) Preservation of pseudotachylyte microstructures

The microstructures described above, including amorphous and/or cryptocrystalline material are clearly inherited from the time interval of dynamic rupture, frictional sliding and cooling towards ambient temperatures. There is no evidence for reactions (including hydration reactions) after the quenching of the frictional melt. Incipient deformation may be recorded in some pseudotachylytes by the elongated shape of clasts (figure 2) and dislocation movement (figure 7*d*). Similarly pristine lower crustal pseudotachylytes have been described from the Musgrave Ranges in Australia [2,35], the Ivrea Zone [4] and Nusfjord East in the Lofoten area [6]. This preservation is remarkable considering the long history of these rocks after their formation, including the uplift to lower P, T-conditions.

Thermal models suggest that the cooling of a thin pseudotachylyte melt to ambient temperature lasts for a maximum of seconds to minutes (e.g. [36,37]). The absence of recrystallization and deformation after the formation of the pseudotachylyte demonstrates that in dry systems at lower crustal temperatures, solid-state nucleation and growth processes are exceedingly slow. Assuming diffusion-controlled growth, a diffusion length below 10 nm (smaller than what we could observe) and a minimum residence time at lower crustal conditions of 1 million years, the associated diffusion coefficient would be below *ca* 10^−30^ m^2^ s^−1^. This value is realistic for CaAl-NaSi interdiffusion in dry systems, even at lower crustal conditions [38].

## Data accessibility

The raw EBSD data that was used to produce figure 5*e,f* can be accessed here: https://osf.io/peyn6.

## References

[1] Sibson RH, Toy VG 2006 The habitat of fault-generated pseudotachylyte: Presence vs. absence of friction-melt. In Earthquakes: radiated energy and the physics of faulting. Geophysical monograph, vol. 170 (eds R Abercrombie, A McGarr, H Kanamori, G Di Toro), pp. 153–166. Washington, DC: American Geophysical Union.

[2] Hawemann F, Mancktelow NS, Wex S, Camacho A, Pennacchioni G 2018 Pseudotachylyte as field evidence for lower-crustal earthquakes during the intracontinental Petermann Orogeny (Musgrave Block, Central Australia). Solid Earth 9, 629–648. (10.5194/se-9-629-2018)

[3] Orlandini OF, Mahan KH, Williams MJ, Regan SP, Mueller KJ 2019 Evidence for deep crustal seismic rupture in a granulite-facies, intraplate, strike-slip shear zone, northern Saskatchewan, Canada. GSA Bullet. 131, 403–425. (10.1130/B31922.1)

[4] Pittarello L, Pennacchioni G, Di Toro G. 2012 Amphibolite-facies pseudotachylytes in Premosello metagabbro and felsic mylonites (Ivrea Zone, Italy). Tectonophysics 580, 43–57. (10.1016/j.tecto.2012.08.001)

[5] Steltenpohl MG, Kassos G, Andresen A 2006 Retrograded eclogite-facies pseudotachylytes as deep-crustal paleoseismic faults within continental basement of Lofoten, north Norway. Geosphere 2, 61–72. (10.1130/ges00035.1)

[6] Menegon L, Pennacchioni G, Malaspina N, Harris K, Wood E 2017 Earthquakes as precursors of ductile shear zones in the dry and strong lower crust. Geochem. Geophys. Geosyst. 18, 4356–4374. (10.1002/2017GC007189)

[7] Jamtveit B, Petley-Ragan A, Incel S, Dunkel KG, Aupart C, Austrheim H, Corfu F, Menegon L, Renard F 2019 The effects of earthquakes and fluids on the metamorphism of the lower continental crust. J. Geophys. Res. Solid Earth 124, 7725–7755. (10.1029/2018jb016461)

[8] Clarke G, Norman A 1993 Generation of pseudotachylite under granulite facies conditions, and its preservation during cooling. J. Metamorph. Geol. 11, 319–335. (10.1111/j.1525-1314.1993.tb00151.x)

[9] White JC 2012 Paradoxical pseudotachylyte – Fault melt outside the seismogenic zone. J. Struct. Geol. 38, 11–20. (10.1016/j.jsg.2011.11.016)

[10] Jackson J, McKenzie D, Priestley K, Emmerson B 2008 New views on the structure and rheology of the lithosphere. J. Geol. Soc. 165, 453–465. (10.1144/0016-76492007-109)

[11] Jamtveit B, Ben-Zion Y, Renard F, Austrheim H 2018 Earthquake-induced transformation of the lower crust. Nature 556, 487–491. (10.1038/s41586-018-0045-y) 29695846 PMC5935234

[12] Braeck S, Podladchikov YY 2007 Spontaneous thermal runaway as an ultimate failure mechanism of materials. Phys. Rev. Lett. 98, 095504 (10.1103/PhysRevLett.98.095504) 17359169

[13] John T, Medvedev S, Rüpke LH, Andersen TB, Podladchikov YY, Austrheim H 2009 Generation of intermediate-depth earthquakes by self-localizing thermal runaway. Nat. Geosci. 2, 137–140. (10.1038/ngeo419)

[14] Hobbs BE, Ord A 1988 Plastic instabilities: Implications for the origin of intermediate and deep focus earthquakes. J. Geophys. Res. Solid Earth 93, 10 521–10 540. (10.1029/JB093iB09p10521)

[15] Austrheim H, Boundy TM 1994 Pseudotachylytes generated during seismic faulting and eclogitization of the deep crust. Science 265, 82–83. (10.1126/science.265.5168.82) 17774692

[16] Kirkpatrick JD, Rowe CD 2013 Disappearing ink: how pseudotachylytes are lost from the rock record. J. Struct. Geol. 52, 183–198. (10.1016/j.jsg.2013.03.003)

[17] Dunkel KG, Zhong X, Arnestad PF, Valen LV, Jamtveit B In press High transient stress in the lower crust: evidence from dry pseudotachylytes in granulites, Lofoten Archipelago, Northern Norway. Geology. (10.1130/G48002.1)

[18] Bachmann F, Hielscher R, Schaeben H 2010 Texture analysis with MTEX–free and open source software toolbox. Solid State Phenomena 160, 63–68.

[19] Bachmann F, Hielscher R, Schaeben H 2011 Grain detection from 2d and 3d EBSD data—Specification of the MTEX algorithm. Ultramicroscopy 111, 1720–1733. (10.1016/j.ultramic.2011.08.002) 22094374

[20] Hielscher R, Schaeben H 2008 A novel pole figure inversion method: specification of the MTEX algorithm. J. Appl. Crystallogr. 41, 1024–1037. (10.1107/S0021889808030112)

[21] Corfu F 2004 U–Pb Age, Setting and Tectonic Significance of the Anorthosite–Mangerite–Charnockite–Granite Suite, Lofoten–Vesterålen, Norway. J. Petrol. 45, 1799–1819. (10.1093/petrology/egh034)

[22] Griffin W, Taylor P, Hakkinen J, Heier K, Iden I, Krogh E, Malm O, Olsen K, Ormaasen D, Tveten E 1978 Archaean and proterozoic crustal evolution in Lofoten–Vesterålen, N Norway. J. Geol. Soc. 135, 629–647. (10.1144/gsjgs.135.6.0629)

[23] Steltenpohl MG, Hames WE, Andresen A 2004 The Silurian to Permian history of a metamorphic core complex in Lofoten, northern Scandinavian Caledonides. Tectonics 23 (10.1029/2003tc001522)

[24] Moecher D, Steltenpohl M 2009 Direct calculation of rupture depth for an exhumed paleoseismogenic fault from mylonitic pseudotachylyte. Geology 37, 999–1002. (10.1130/G30166A.1)

[25] Moecher DP, Steltenpohl MG 2011 Petrological evidence for co-seismic slip in extending middle–lower continental crust: Heier's zone of pseudotachylyte, north Norway. Geol. Soc. Lond. Spec. Pub. 359, 169–186. (10.1144/sp359.10)

[26] Steltenpohl MG, Moecher D, Andresen A, Ball J, Mager S, Hames WE 2011 The Eidsfjord shear zone, Lofoten–Vesterålen, north Norway: An Early Devonian, paleoseismogenic low-angle normal fault. J. Struct. Geol. 33, 1023–1043. (10.1016/j.jsg.2011.01.017)

[27] Leib S, Moecher D, Steltenpohl M, Andresen A 2016 Thermobarometry of metamorphosed pseudotachylyte and associated mylonite: Constraints on dynamic Co-seismic rupture depth attending Caledonian extension, North Norway. Tectonophysics 682, 85–95. (10.1016/j.tecto.2016.05.042)

[28] Plattner U, Markl G, Sherlock S 2003 Chemical heterogeneities of Caledonian (?) pseudotachylites in the Eidsfjord Anorthosite, north Norway. Contrib. Mineral. Petrol. 145, 316–338. (10.1007/s00410-003-0455-0)

[29] Lin A 2007 Fossil earthquakes: The formation and preservation of pseudotachylytes. Berlin, Germany: Springer.

[30] Gránásy L, Pusztai T, Tegze G, Warren JA, Douglas JF 2005 Growth and form of spherulites. Phys. Rev. E 72, 011605 (10.1103/PhysRevE.72.011605) 16089977

[31] Lofgren G, Hargraves R 1980 Experimental studies on the dynamic crystallization of silicate melts. Phys. mag. process. 487, 551.

[32] Kirkpatrick RJ 1975 Crystal growth from the melt: a review. Am. Mineral. J. Earth Planet. Mat. 60, 798–814.

[33] Lofgren G 1974 An experimental study of plagioclase crystal morphology; isothermal crystallization. Am. J. Sci. 274, 243–273. (10.2475/ajs.274.3.243)

[34] Di Toro G, Pennacchioni G. 2004 Superheated friction-induced melts in zoned pseudotachylytes within the Adamello tonalites (Italian Southern Alps). J. Struct. Geol. 26, 1783–1801. (10.1016/j.jsg.2004.03.001)

[35] Camacho A, Vernon R, Gerald JF 1995 Large volumes of anhydrous pseudotachylyte in the Woodroffe Thrust, eastern Musgrave Ranges, Australia. J. Struct. Geol. 17, 371–383. (10.1016/0191-8141(94)00069-C)

[36] Clerc A, Renard F, Austrheim H, Jamtveit B 2018 Spatial and size distributions of garnets grown in a pseudotachylyte generated during a lower crust earthquake. Tectonophysics 733, 159–170. (10.1016/j.tecto.2018.02.014)

[37] Bestmann M, Pennacchioni G, Nielsen S, Göken M, de Wall H. 2012 Deformation and ultrafine dynamic recrystallization of quartz in pseudotachylyte-bearing brittle faults: A matter of a few seconds. J. Struct. Geol. 38, 21–38. (10.1016/j.jsg.2011.10.001)

[38] Cherniak D 2010 Cation diffusion in feldspars. Rev. Mineral. Geochem. 72, 691–733.

